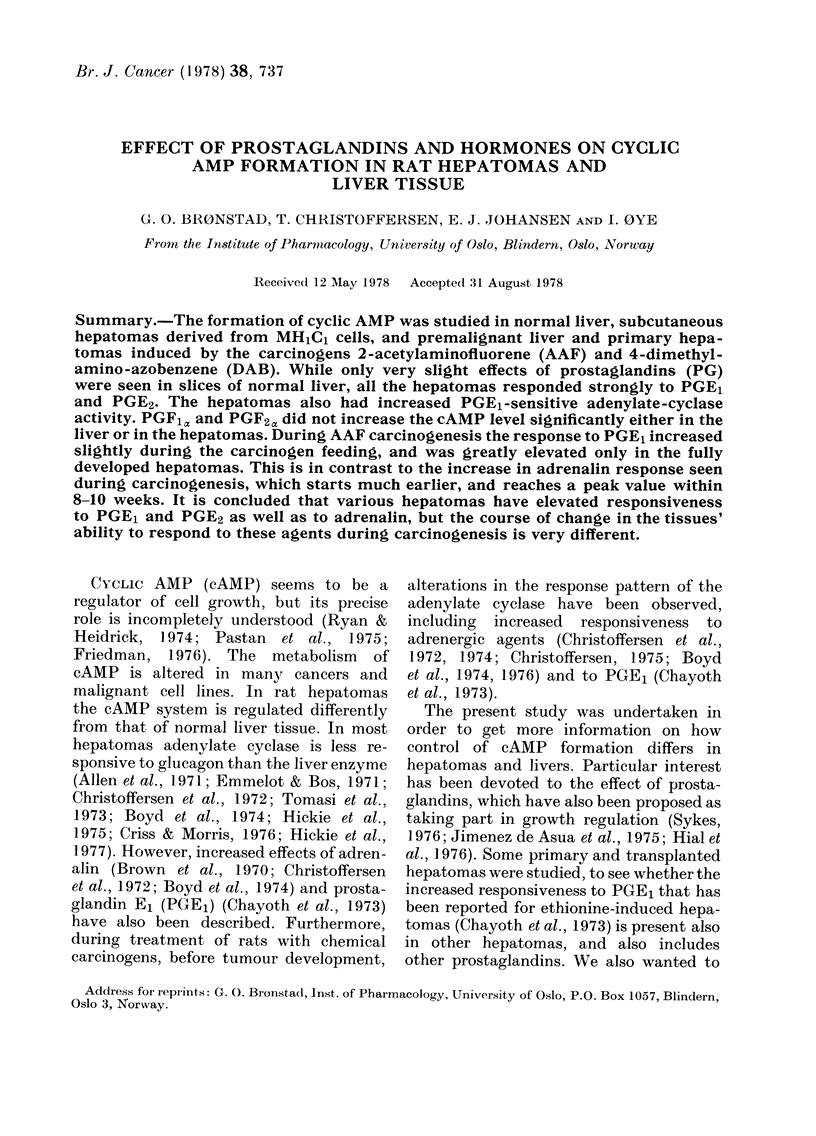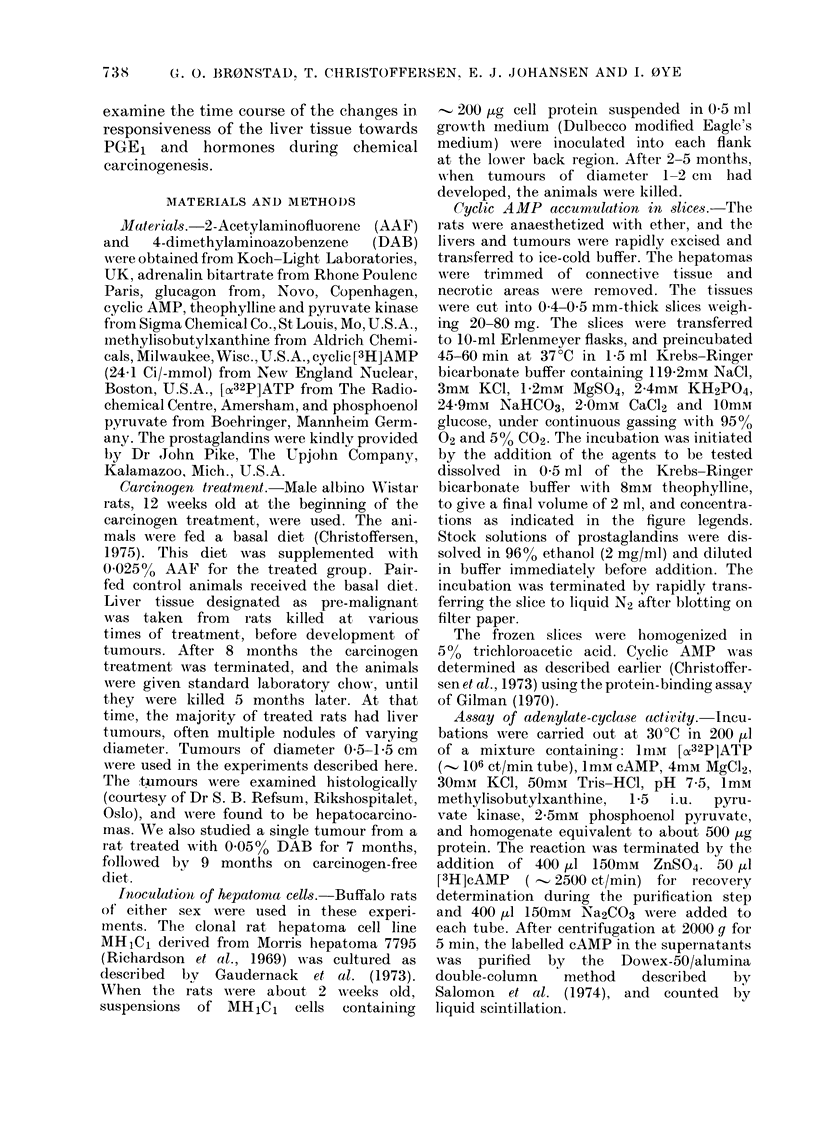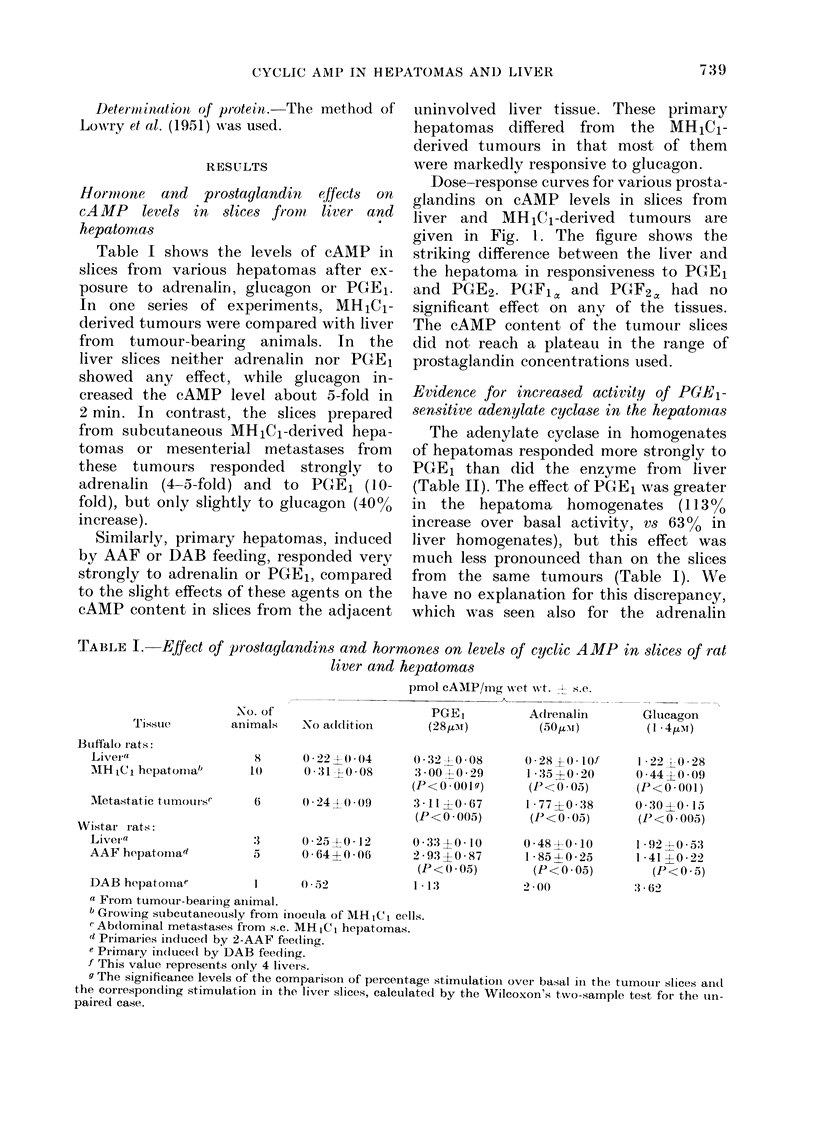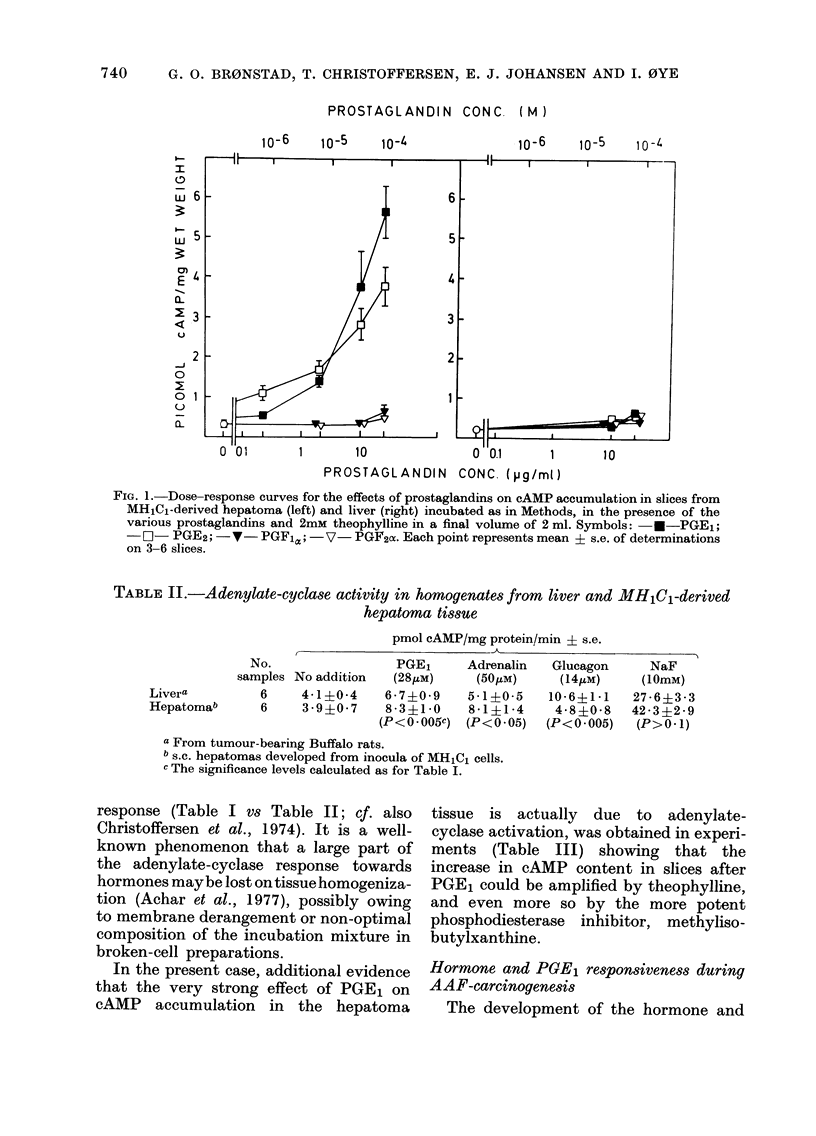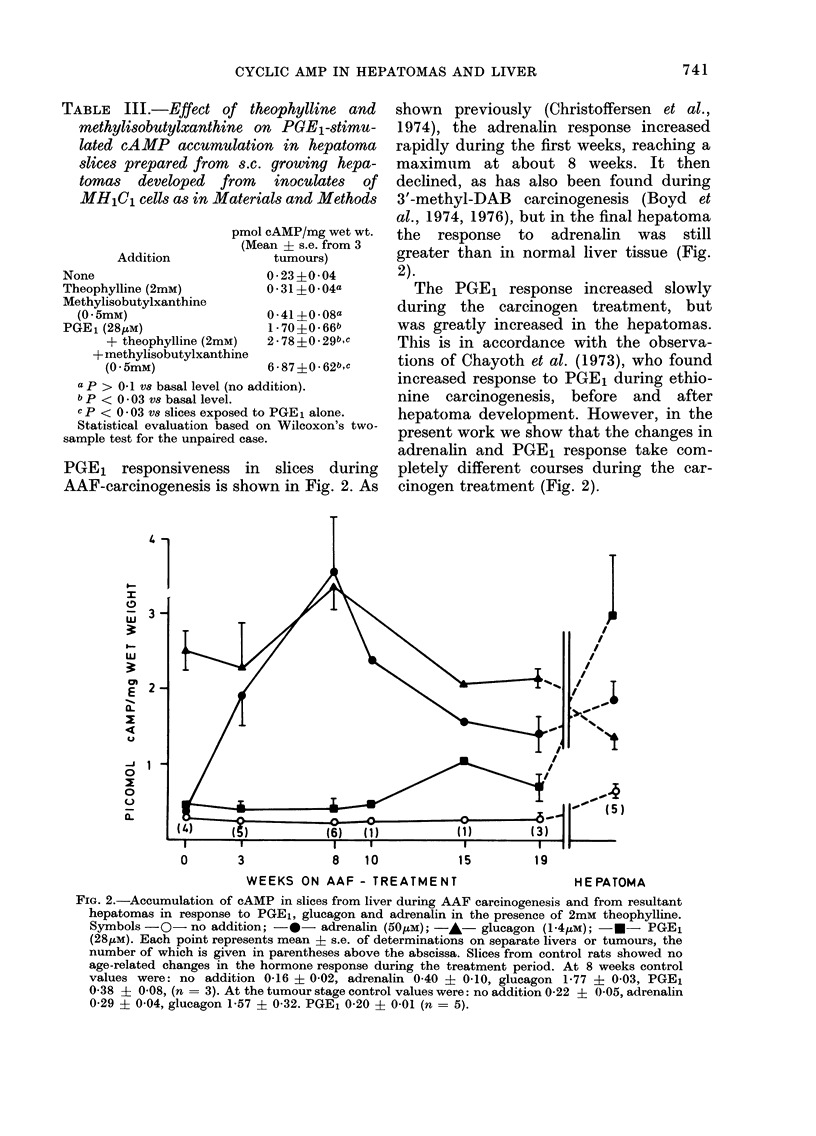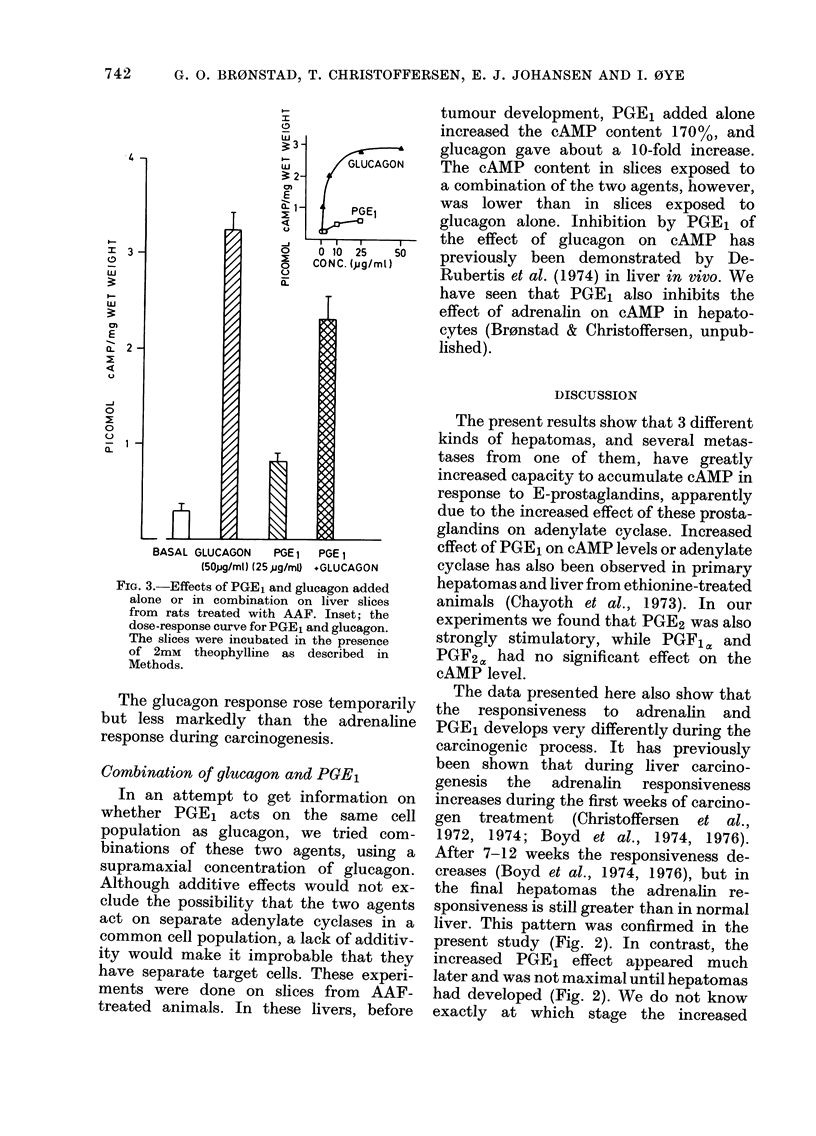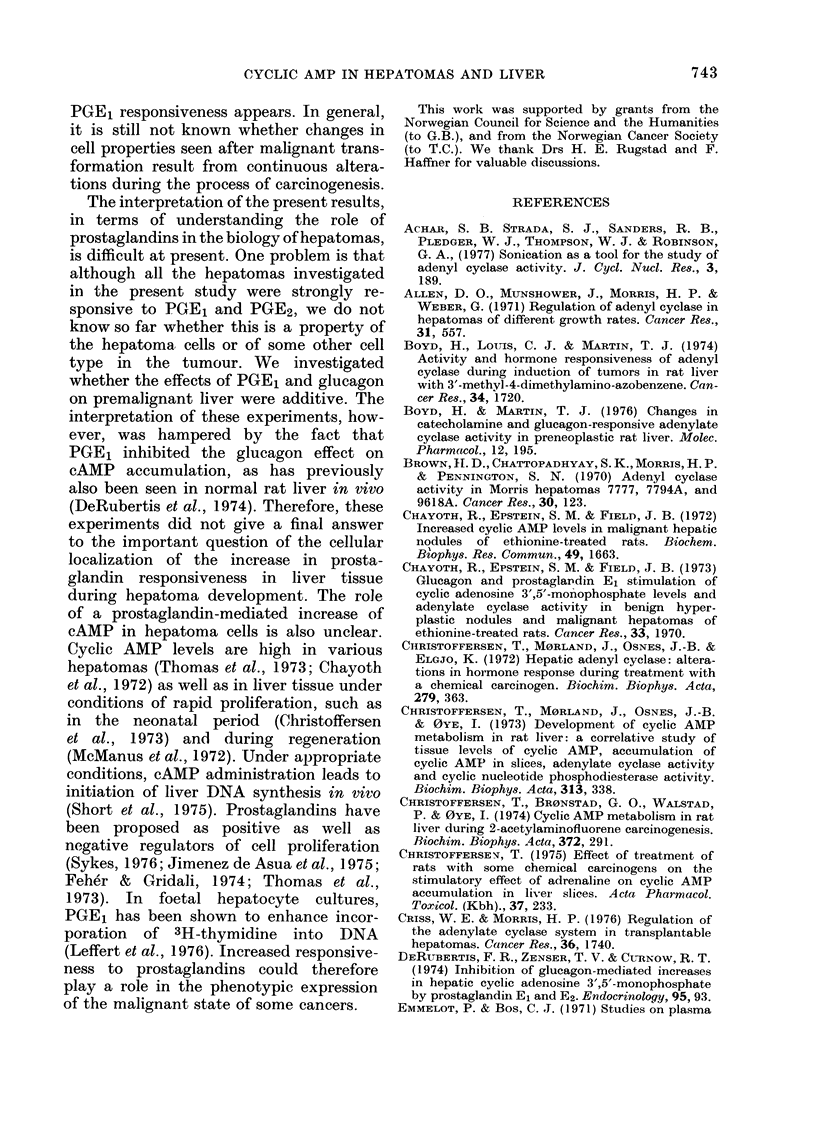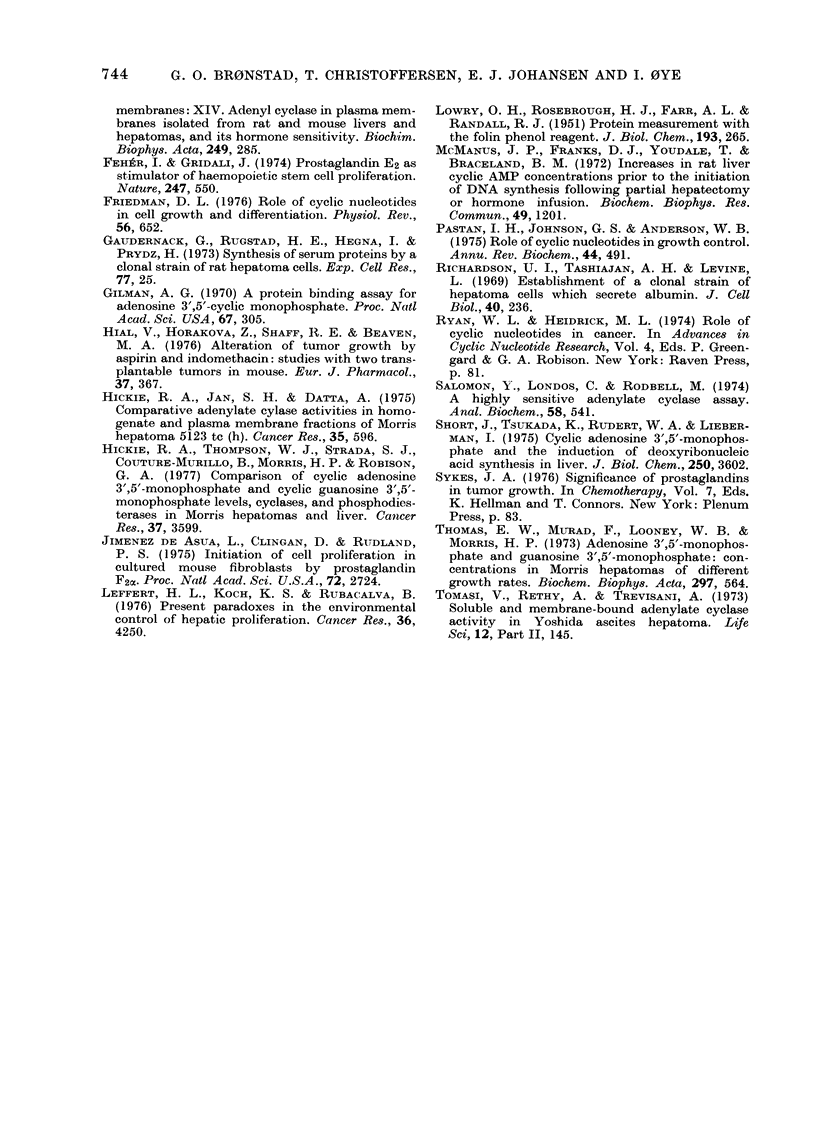# Effect of prostaglandins and hormones on cyclic AMP formation in rat hepatomas and liver tissue.

**DOI:** 10.1038/bjc.1978.281

**Published:** 1978-12

**Authors:** G. O. Brønstad, T. Christoffersen, E. J. Johansen, I. Oye

## Abstract

The formation of cyclic AMP was studied in normal liver, subcutaneous hepatomas derived from MH1C1 cells, and premalignant liver and primary hepatomas induced by the carcinogens 2-acetylaminofluorene (AAF) and 4-dimethylamino-azobenzene (DAB). While only very slight effects of prostaglandins (PG) were seen in slices of normal liver, all the hepatomas responded strongly to PGE1 and PGE2. The hepatomas also had increase PGE1-sensitive adenylate-cyclase activity. PGF1alpha and PGF2alpha did not increase the cAMP level significantly either in the liver or in the hepatomas. During AAF carcinogenesis the response to PGE1 increased slightly during the carcinogen feeding, and was greatly elevated only in the fully developed hepatomas. This is in contrast to the increase in adrenalin response seen during carcinogenesis, which starts much earlier, and reaches a peak value within 8--10 weeks. It is concluded that various hepatomas have elevated responsiveness to PGE1 and PGE2 as well as to adrenalin, but the course of change in the tissues' ability to respond to these agents during carcinogenesis is very different.


*Br. J. Cancer* (1978) **38**, 737

### EFFECT OF PROSTAGLANDINS AND HORMONES ON CYCLIC AMP FORMATION IN RAT HEPATOMAS AND LIVER TISSUE

G. O. BRØNSTAD, T. CHRISTOFFERSEN, E. J. JOHANSEN AND I. ØYE

*From the Institute of Pharmacology, University of Oslo, Blindern, Oslo, Norway*

Received 12 May 1978 Accepted 31 August 1978

**Summary.—The formation of cyclic AMP was studied in normal liver, subcutaneous hepatomas derived from $MH_1C_1$ cells, and premalignant liver and primary hepatomas induced by the carcinogens 2-acetylaminofluorene (AAF) and 4-dimethylamino-azobenzene (DAB). While only very slight effects of prostaglandins (PG) were seen in slices of normal liver, all the hepatomas responded strongly to $PGE_1$ and $PGE_2$. The hepatomas also had increased $PGE_1$-sensitive adenylate-cyclase activity. $PGF_{1\alpha}$ and $PGF_{2\alpha}$ did not increase the cAMP level significantly either in the liver or in the hepatomas. During AAF carcinogenesis the response to $PGE_1$ increased slightly during the carcinogen feeding, and was greatly elevated only in the fully developed hepatomas. This is in contrast to the increase in adrenalin response seen during carcinogenesis, which starts much earlier, and reaches a peak value within 8–10 weeks. It is concluded that various hepatomas have elevated responsiveness to $PGE_1$ and $PGE_2$ as well as to adrenalin, but the course of change in the tissues' ability to respond to these agents during carcinogenesis is very different.**

Cyclic AMP (cAMP) seems to be a regulator of cell growth, but its precise role is incompletely understood (Ryan & Heidrick, 1974; Pastan *et al.*, 1975; Friedman, 1976). The metabolism of cAMP is altered in many cancers and malignant cell lines. In rat hepatomas the cAMP system is regulated differently from that of normal liver tissue. In most hepatomas adenylate cyclase is less responsive to glucagon than the liver enzyme (Allen *et al.*, 1971; Emmelot & Bos, 1971; Christoffersen *et al.*, 1972; Tomasi *et al.*, 1973; Boyd *et al.*, 1974; Hickie *et al.*, 1975; Criss & Morris, 1976; Hickie *et al.*, 1977). However, increased effects of adrenalin (Brown *et al.*, 1970; Christoffersen *et al.*, 1972; Boyd *et al.*, 1974) and prostaglandin $E_1$ ($PGE_1$) (Chayoth *et al.*, 1973) have also been described. Furthermore, during treatment of rats with chemical carcinogens, before tumour development, alterations in the response pattern of the adenylate cyclase have been observed, including increased responsiveness to adrenergic agents (Christoffersen *et al.*, 1972, 1974; Christoffersen, 1975; Boyd *et al.*, 1974, 1976) and to $PGE_1$ (Chayoth *et al.*, 1973).

The present study was undertaken in order to get more information on how control of cAMP formation differs in hepatomas and livers. Particular interest has been devoted to the effect of prostaglandins, which have also been proposed as taking part in growth regulation (Sykes, 1976; Jimenez de Asua *et al.*, 1975; Hial *et al.*, 1976). Some primary and transplanted hepatomas were studied, to see whether the increased responsiveness to $PGE_1$ that has been reported for ethionine-induced hepatomas (Chayoth *et al.*, 1973) is present also in other hepatomas, and also includes other prostaglandins. We also wanted to

Address for reprints: G. O. Brønstad, Inst. of Pharmacology, University of Oslo, P.O. Box 1057, Blindern, Oslo 3, Norway.

examine the time course of the changes in responsiveness of the liver tissue towards $PGE_1$ and hormones during chemical carcinogenesis.

### MATERIALS AND METHODS

*Materials.*—2-Acetylaminofluorene (AAF) and 4-dimethylaminoazobenzene (DAB) were obtained from Koch–Light Laboratories, UK, adrenalin bitartrate from Rhone Poulenc Paris, glucagon from, Novo, Copenhagen, cyclic AMP, theophylline and pyruvate kinase from Sigma Chemical Co., St Louis, Mo, U.S.A., methylisobutylxanthine from Aldrich Chemicals, Milwaukee, Wisc., U.S.A., cyclic [$^3$H]AMP (24·1 Ci/-mmol) from New England Nuclear, Boston, U.S.A., [$\alpha^{32}$P]ATP from The Radiochemical Centre, Amersham, and phosphoenol pyruvate from Boehringer, Mannheim Germany. The prostaglandins were kindly provided by Dr John Pike, The Upjohn Company, Kalamazoo, Mich., U.S.A.

*Carcinogen treatment.*—Male albino Wistar rats, 12 weeks old at the beginning of the carcinogen treatment, were used. The animals were fed a basal diet (Christoffersen, 1975). This diet was supplemented with 0·025% AAF for the treated group. Pair-fed control animals received the basal diet. Liver tissue designated as pre-malignant was taken from rats killed at various times of treatment, before development of tumours. After 8 months the carcinogen treatment was terminated, and the animals were given standard laboratory chow, until they were killed 5 months later. At that time, the majority of treated rats had liver tumours, often multiple nodules of varying diameter. Tumours of diameter 0·5–1·5 cm were used in the experiments described here. The tumours were examined histologically (courtesy of Dr S. B. Refsum, Rikshospitalet, Oslo), and were found to be hepatocarcinomas. We also studied a single tumour from a rat treated with 0·05% DAB for 7 months, followed by 9 months on carcinogen-free diet.

*Inoculation of hepatoma cells.*—Buffalo rats of either sex were used in these experiments. The clonal rat hepatoma cell line $MH_1C_1$ derived from Morris hepatoma 7795 (Richardson *et al.*, 1969) was cultured as described by Gaudernack *et al.* (1973). When the rats were about 2 weeks old, suspensions of $MH_1C_1$ cells containing $\sim$ 200 $\mu$g cell protein suspended in 0·5 ml growth medium (Dulbecco modified Eagle's medium) were inoculated into each flank at the lower back region. After 2–5 months, when tumours of diameter 1–2 cm had developed, the animals were killed.

*Cyclic AMP accumulation in slices.*—The rats were anaesthetized with ether, and the livers and tumours were rapidly excised and transferred to ice-cold buffer. The hepatomas were trimmed of connective tissue and necrotic areas were removed. The tissues were cut into 0·4–0·5 mm-thick slices weighing 20–80 mg. The slices were transferred to 10-ml Erlenmeyer flasks, and preincubated 45–60 min at 37°C in 1·5 ml Krebs–Ringer bicarbonate buffer containing 119·2mM NaCl, 3mM KCl, 1·2mM $MgSO_4$, 2·4mM $KH_2PO_4$, 24·9mM $NaHCO_3$, 2·0mM $CaCl_2$ and 10mM glucose, under continuous gassing with 95% $O_2$ and 5% $CO_2$. The incubation was initiated by the addition of the agents to be tested dissolved in 0·5 ml of the Krebs–Ringer bicarbonate buffer with 8mM theophylline, to give a final volume of 2 ml, and concentrations as indicated in the figure legends. Stock solutions of prostaglandins were dissolved in 96% ethanol (2 mg/ml) and diluted in buffer immediately before addition. The incubation was terminated by rapidly transferring the slice to liquid $N_2$ after blotting on filter paper.

The frozen slices were homogenized in 5% trichloroacetic acid. Cyclic AMP was determined as described earlier (Christoffersen *et al.*, 1973) using the protein-binding assay of Gilman (1970).

*Assay of adenylate-cyclase activity.*—Incubations were carried out at 30°C in 200 $\mu$l of a mixture containing: 1mM [$\alpha^{32}$P]ATP ($\sim 10^6$ ct/min tube), 1mM cAMP, 4mM $MgCl_2$, 30mM KCl, 50mM Tris–HCl, pH 7·5, 1mM methylisobutylxanthine, 1·5 i.u. pyruvate kinase, 2·5mM phosphoenol pyruvate, and homogenate equivalent to about 500 $\mu$g protein. The reaction was terminated by the addition of 400 $\mu$l 150mM $ZnSO_4$. 50 $\mu$l [$^3$H]cAMP ( $\sim$ 2500 ct/min) for recovery determination during the purification step and 400 $\mu$l 150mM $Na_2CO_3$ were added to each tube. After centrifugation at 2000 *g* for 5 min, the labelled cAMP in the supernatants was purified by the Dowex-50/alumina double-column method described by Salomon *et al.* (1974), and counted by liquid scintillation.

*Determination of protein.*—The method of Lowry *et al.* (1951) was used.

RESULTS

*Hormone and prostaglandin effects on cAMP levels in slices from liver and hepatomas*

Table I shows the levels of cAMP in slices from various hepatomas after exposure to adrenalin, glucagon or $PGE_1$. In one series of experiments, $MH_1C_1$-derived tumours were compared with liver from tumour-bearing animals. In the liver slices neither adrenalin nor $PGE_1$ showed any effect, while glucagon increased the cAMP level about 5-fold in 2 min. In contrast, the slices prepared from subcutaneous $MH_1C_1$-derived hepatomas or mesenterial metastases from these tumours responded strongly to adrenalin (4–5-fold) and to $PGE_1$ (10-fold), but only slightly to glucagon (40% increase).

Similarly, primary hepatomas, induced by AAF or DAB feeding, responded very strongly to adrenalin or $PGE_1$, compared to the slight effects of these agents on the cAMP content in slices from the adjacent uninvolved liver tissue. These primary hepatomas differed from the $MH_1C_1$-derived tumours in that most of them were markedly responsive to glucagon.

Dose–response curves for various prostaglandins on cAMP levels in slices from liver and $MH_1C_1$-derived tumours are given in Fig. 1. The figure shows the striking difference between the liver and the hepatoma in responsiveness to $PGE_1$ and $PGE_2$. $PGF_{1\alpha}$ and $PGF_{2\alpha}$ had no significant effect on any of the tissues. The cAMP content of the tumour slices did not reach a plateau in the range of prostaglandin concentrations used.

*Evidence for increased activity of $PGE_1$-sensitive adenylate cyclase in the hepatomas*

The adenylate cyclase in homogenates of hepatomas responded more strongly to $PGE_1$ than did the enzyme from liver (Table II). The effect of $PGE_1$ was greater in the hepatoma homogenates (113% increase over basal activity, *vs* 63% in liver homogenates), but this effect was much less pronounced than on the slices from the same tumours (Table I). We have no explanation for this discrepancy, which was seen also for the adrenalin

TABLE I.—*Effect of prostaglandins and hormones on levels of cyclic AMP in slices of rat liver and hepatomas*

| Tissue | No. of animals | pmol cAMP/mg wet wt. ± s.e. No addition | $PGE_1$ (28μM) | Adrenalin (50μM) | Glucagon (1·4μM) |
|---|---|---|---|---|---|
| Buffalo rats: | | | | | |
| Liver[a] | 8 | 0·22±0·04 | 0·32±0·08 | 0·28±0·10[f] | 1·22±0·28 |
| $MH_1C_1$ hepatoma[b] | 10 | 0·31±0·08 | 3·00±0·29 ($P<0·001$[g]) | 1·35±0·20 ($P<0·05$) | 0·44±0·09 ($P<0·001$) |
| Metastatic tumours[c] | 6 | 0·24±0·09 | 3·11±0·67 ($P<0·005$) | 1·77±0·38 ($P<0·05$) | 0·30±0·15 ($P<0·005$) |
| Wistar rats: | | | | | |
| Liver[a] | 3 | 0·25±0·12 | 0·33±0·10 | 0·48±0·10 | 1·92±0·53 |
| AAF hepatoma[d] | 5 | 0·64±0·06 | 2·93±0·87 ($P<0·05$) | 1·85±0·25 ($P<0·05$) | 1·41±0·22 ($P<0·5$) |
| DAB hepatoma[e] | 1 | 0·52 | 1·13 | 2·00 | 3·62 |

[a] From tumour-bearing animal.
[b] Growing subcutaneously from inocula of $MH_1C_1$ cells.
[c] Abdominal metastases from s.c. $MH_1C_1$ hepatomas.
[d] Primaries induced by 2-AAF feeding.
[e] Primary induced by DAB feeding.
[f] This value represents only 4 livers.
[g] The significance levels of the comparison of percentage stimulation over basal in the tumour slices and the corresponding stimulation in the liver slices, calculated by the Wilcoxon's two-sample test for the unpaired case.

FIG. 1.—Dose–response curves for the effects of prostaglandins on cAMP accumulation in slices from $MH_1C_1$-derived hepatoma (left) and liver (right) incubated as in Methods, in the presence of the various prostaglandins and 2mM theophylline in a final volume of 2 ml. Symbols: —■—$PGE_1$; —□— $PGE_2$; —▼— $PGF_{1\alpha}$; —▽— $PGF_{2\alpha}$. Each point represents mean ± s.e. of determinations on 3–6 slices.

TABLE II.—*Adenylate-cyclase activity in homogenates from liver and $MH_1C_1$-derived hepatoma tissue*

| | No. samples | pmol cAMP/mg protein/min ± s.e. No addition | $PGE_1$ (28μM) | Adrenalin (50μM) | Glucagon (14μM) | NaF (10mM) |
|---|---|---|---|---|---|---|
| Liver[a] | 6 | 4·1±0·4 | 6·7±0·9 | 5·1±0·5 | 10·6±1·1 | 27·6±3·3 |
| Hepatoma[b] | 6 | 3·9±0·7 | 8·3±1·0 | 8·1±1·4 | 4·8±0·8 | 42·3±2·9 |
| | | | ($P<0·005$[c]) | ($P<0·05$) | ($P<0·005$) | ($P>0·1$) |

[a] From tumour-bearing Buffalo rats.
[b] s.c. hepatomas developed from inocula of $MH_1C_1$ cells.
[c] The significance levels calculated as for Table I.

response (Table I *vs* Table II; *cf.* also Christoffersen *et al.*, 1974). It is a well-known phenomenon that a large part of the adenylate-cyclase response towards hormones may be lost on tissue homogenization (Achar *et al.*, 1977), possibly owing to membrane derangement or non-optimal composition of the incubation mixture in broken-cell preparations.

In the present case, additional evidence that the very strong effect of $PGE_1$ on cAMP accumulation in the hepatoma tissue is actually due to adenylate-cyclase activation, was obtained in experiments (Table III) showing that the increase in cAMP content in slices after $PGE_1$ could be amplified by theophylline, and even more so by the more potent phosphodiesterase inhibitor, methylisobutylxanthine.

### *Hormone and $PGE_1$ responsiveness during AAF-carcinogenesis*

The development of the hormone and

TABLE III.—*Effect of theophylline and methylisobutylxanthine on $PGE_1$-stimulated cAMP accumulation in hepatoma slices prepared from s.c. growing hepatomas developed from inoculates of $MH_1C_1$ cells as in Materials and Methods*

| Addition | pmol cAMP/mg wet wt. (Mean ± s.e. from 3 tumours) |
|---|---|
| None | 0·23±0·04 |
| Theophylline (2mM) | 0·31±0·04[a] |
| Methylisobutylxanthine (0·5mM) | 0·41±0·08[a] |
| $PGE_1$ (28μM) | 1·70±0·66[b] |
| + theophylline (2mM) | 2·78±0·29[b,c] |
| +methylisobutylxanthine (0·5mM) | 6·87±0·62[b,c] |

[a] $P > 0·1$ *vs* basal level (no addition).
[b] $P < 0·03$ *vs* basal level.
[c] $P < 0·03$ *vs* slices exposed to $PGE_1$ alone.
Statistical evaluation based on Wilcoxon's two-sample test for the unpaired case.

$PGE_1$ responsiveness in slices during AAF-carcinogenesis is shown in Fig. 2. As shown previously (Christoffersen *et al.*, 1974), the adrenalin response increased rapidly during the first weeks, reaching a maximum at about 8 weeks. It then declined, as has also been found during 3′-methyl-DAB carcinogenesis (Boyd *et al.*, 1974, 1976), but in the final hepatoma the response to adrenalin was still greater than in normal liver tissue (Fig. 2).

The $PGE_1$ response increased slowly during the carcinogen treatment, but was greatly increased in the hepatomas. This is in accordance with the observations of Chayoth *et al.* (1973), who found increased response to $PGE_1$ during ethionine carcinogenesis, before and after hepatoma development. However, in the present work we show that the changes in adrenalin and $PGE_1$ response take completely different courses during the carcinogen treatment (Fig. 2).

FIG. 2.—Accumulation of cAMP in slices from liver during AAF carcinogenesis and from resultant hepatomas in response to $PGE_1$, glucagon and adrenalin in the presence of 2mM theophylline. Symbols —○— no addition; —●— adrenalin (50μM); —▲— glucagon (1·4μM); —■— $PGE_1$ (28μM). Each point represents mean ± s.e. of determinations on separate livers or tumours, the number of which is given in parentheses above the abscissa. Slices from control rats showed no age-related changes in the hormone response during the treatment period. At 8 weeks control values were: no addition 0·16 ± 0·02, adrenalin 0·40 ± 0·10, glucagon 1·77 ± 0·03, $PGE_1$ 0·38 ± 0·08, ($n$ = 3). At the tumour stage control values were: no addition 0·22 ± 0·05, adrenalin 0·29 ± 0·04, glucagon 1·57 ± 0·32. $PGE_1$ 0·20 ± 0·01 ($n$ = 5).

FIG. 3.—Effects of $PGE_1$ and glucagon added alone or in combination on liver slices from rats treated with AAF. Inset; the dose-response curve for $PGE_1$ and glucagon. The slices were incubated in the presence of 2mM theophylline as described in Methods.

The glucagon response rose temporarily but less markedly than the adrenaline response during carcinogenesis.

*Combination of glucagon and $PGE_1$*

In an attempt to get information on whether $PGE_1$ acts on the same cell population as glucagon, we tried combinations of these two agents, using a supramaxial concentration of glucagon. Although additive effects would not exclude the possibility that the two agents act on separate adenylate cyclases in a common cell population, a lack of additivity would make it improbable that they have separate target cells. These experiments were done on slices from AAF-treated animals. In these livers, before tumour development, $PGE_1$ added alone increased the cAMP content 170%, and glucagon gave about a 10-fold increase. The cAMP content in slices exposed to a combination of the two agents, however, was lower than in slices exposed to glucagon alone. Inhibition by $PGE_1$ of the effect of glucagon on cAMP has previously been demonstrated by DeRubertis *et al.* (1974) in liver *in vivo*. We have seen that $PGE_1$ also inhibits the effect of adrenalin on cAMP in hepatocytes (Brønstad & Christoffersen, unpublished).

### DISCUSSION

The present results show that 3 different kinds of hepatomas, and several metastases from one of them, have greatly increased capacity to accumulate cAMP in response to E-prostaglandins, apparently due to the increased effect of these prostaglandins on adenylate cyclase. Increased effect of $PGE_1$ on cAMP levels or adenylate cyclase has also been observed in primary hepatomas and liver from ethionine-treated animals (Chayoth *et al.*, 1973). In our experiments we found that $PGE_2$ was also strongly stimulatory, while $PGF_{1\alpha}$ and $PGF_{2\alpha}$ had no significant effect on the cAMP level.

The data presented here also show that the responsiveness to adrenalin and $PGE_1$ develops very differently during the carcinogenic process. It has previously been shown that during liver carcinogenesis the adrenalin responsiveness increases during the first weeks of carcinogen treatment (Christoffersen *et al.*, 1972, 1974; Boyd *et al.*, 1974, 1976). After 7–12 weeks the responsiveness decreases (Boyd *et al.*, 1974, 1976), but in the final hepatomas the adrenalin responsiveness is still greater than in normal liver. This pattern was confirmed in the present study (Fig. 2). In contrast, the increased $PGE_1$ effect appeared much later and was not maximal until hepatomas had developed (Fig. 2). We do not know exactly at which stage the increased

$PGE_1$ responsiveness appears. In general, it is still not known whether changes in cell properties seen after malignant transformation result from continuous alterations during the process of carcinogenesis.

The interpretation of the present results, in terms of understanding the role of prostaglandins in the biology of hepatomas, is difficult at present. One problem is that although all the hepatomas investigated in the present study were strongly responsive to $PGE_1$ and $PGE_2$, we do not know so far whether this is a property of the hepatoma cells or of some other cell type in the tumour. We investigated whether the effects of $PGE_1$ and glucagon on premalignant liver were additive. The interpretation of these experiments, however, was hampered by the fact that $PGE_1$ inhibited the glucagon effect on cAMP accumulation, as has previously also been seen in normal rat liver *in vivo* (DeRubertis *et al.*, 1974). Therefore, these experiments did not give a final answer to the important question of the cellular localization of the increase in prostaglandin responsiveness in liver tissue during hepatoma development. The role of a prostaglandin-mediated increase of cAMP in hepatoma cells is also unclear. Cyclic AMP levels are high in various hepatomas (Thomas *et al.*, 1973; Chayoth *et al.*, 1972) as well as in liver tissue under conditions of rapid proliferation, such as in the neonatal period (Christoffersen *et al.*, 1973) and during regeneration (McManus *et al.*, 1972). Under appropriate conditions, cAMP administration leads to initiation of liver DNA synthesis *in vivo* (Short *et al.*, 1975). Prostaglandins have been proposed as positive as well as negative regulators of cell proliferation (Sykes, 1976; Jimenez de Asua *et al.*, 1975; Fehér & Gridali, 1974; Thomas *et al.*, 1973). In foetal hepatocyte cultures, $PGE_1$ has been shown to enhance incorporation of $^3H$-thymidine into DNA (Leffert *et al.*, 1976). Increased responsiveness to prostaglandins could therefore play a role in the phenotypic expression of the malignant state of some cancers.

This work was supported by grants from the Norwegian Council for Science and the Humanities (to G.B.), and from the Norwegian Cancer Society (to T.C.). We thank Drs H. E. Rugstad and F. Haffner for valuable discussions.

### REFERENCES

ACHAR, S. B. STRADA, S. J., SANDERS, R. B., PLEDGER, W. J., THOMPSON, W. J. & ROBINSON, G. A., (1977) Sonication as a tool for the study of adenyl cyclase activity. *J. Cycl. Nucl. Res.*, **3**, 189.

ALLEN, D. O., MUNSHOWER, J., MORRIS, H. P. & WEBER, G. (1971) Regulation of adenyl cyclase in hepatomas of different growth rates. *Cancer Res.*, **31**, 557.

BOYD, H., LOUIS, C. J. & MARTIN, T. J. (1974) Activity and hormone responsiveness of adenyl cyclase during induction of tumors in rat liver with 3′-methyl-4-dimethylamino-azobenzene. *Cancer Res.*, **34**, 1720.

BOYD, H. & MARTIN, T. J. (1976) Changes in catecholamine and glucagon-responsive adenylate cyclase activity in preneoplastic rat liver. *Molec. Pharmacol.*, 12, 195.

BROWN, H. D., CHATTOPADHYAY, S. K., MORRIS, H. P. & PENNINGTON, S. N. (1970) Adenyl cyclase activity in Morris hepatomas 7777, 7794A, and 9618A. *Cancer Res.*, **30**, 123.

CHAYOTH, R., EPSTEIN, S. M. & FIELD, J. B. (1972) Increased cyclic AMP levels in malignant hepatic nodules of ethionine-treated rats. *Biochem. Biophys. Res. Commun.*, **49**, 1663.

CHAYOTH, R., EPSTEIN, S. M. & FIELD, J. B. (1973) Glucagon and prostaglandin $E_1$ stimulation of cyclic adenosine 3′,5′-monophosphate levels and adenylate cyclase activity in benign hyperplastic nodules and malignant hepatomas of ethionine-treated rats. *Cancer Res.*, **33**, 1970.

CHRISTOFFERSEN, T., MØRLAND, J., OSNES, J.-B. & ELGJO, K. (1972) Hepatic adenyl cyclase: alterations in hormone response during treatment with a chemical carcinogen. *Biochim. Biophys. Acta*, **279**, 363.

CHRISTOFFERSEN, T., MØRLAND, J., OSNES, J.-B. & ØYE, I. (1973) Development of cyclic AMP metabolism in rat liver: a correlative study of tissue levels of cyclic AMP, accumulation of cyclic AMP in slices, adenylate cyclase activity and cyclic nucleotide phosphodiesterase activity. *Biochim. Biophys. Acta*, **313**, 338.

CHRISTOFFERSEN, T., BRØNSTAD, G. O., WALSTAD, P. & ØYE, I. (1974) Cyclic AMP metabolism in rat liver during 2-acetylaminofluorene carcinogenesis. *Biochim. Biophys. Acta*, **372**, 291.

CHRISTOFFERSEN, T. (1975) Effect of treatment of rats with some chemical carcinogens on the stimulatory effect of adrenaline on cyclic AMP accumulation in liver slices. *Acta Pharmacol. Toxicol.* (Kbh)., **37**, 233.

CRISS, W. E. & MORRIS, H. P. (1976) Regulation of the adenylate cyclase system in transplantable hepatomas. *Cancer Res.*, **36**, 1740.

DERUBERTIS, F. R., ZENSER, T. V. & CURNOW, R. T. (1974) Inhibition of glucagon-mediated increases in hepatic cyclic adenosine 3′,5′-monophosphate by prostaglandin $E_1$ and $E_2$. *Endocrinology*, **95**, 93.

EMMELOT, P. & BOS, C. J. (1971) Studies on plasma membranes: XIV. Adenyl cyclase in plasma membranes isolated from rat and mouse livers and hepatomas, and its hormone sensitivity. *Biochim. Biophys. Acta*, **249**, 285.

FEHÉR, I. & GRIDALI, J. (1974) Prostaglandin $E_2$ as stimulator of haemopoietic stem cell proliferation. *Nature*, **247**, 550.

FRIEDMAN, D. L. (1976) Role of cyclic nucleotides in cell growth and differentiation. *Physiol. Rev.*, **56**, 652.

GAUDERNACK, G., RUGSTAD, H. E., HEGNA, I. & PRYDZ, H. (1973) Synthesis of serum proteins by a clonal strain of rat hepatoma cells. *Exp. Cell Res.*, **77**, 25.

GILMAN, A. G. (1970) A protein binding assay for adenosine 3′,5′-cyclic monophosphate. *Proc. Natl Acad. Sci. USA*, **67**, 305.

HIAL, V., HORAKOVA, Z., SHAFF, R. E. & BEAVEN, M. A. (1976) Alteration of tumor growth by aspirin and indomethacin: studies with two transplantable tumors in mouse. *Eur. J. Pharmacol.*, **37**, 367.

HICKIE, R. A., JAN, S. H. & DATTA, A. (1975) Comparative adenylate cylase activities in homogenate and plasma membrane fractions of Morris hepatoma 5123 tc (h). *Cancer Res.*, **35**, 596.

HICKIE, R. A., THOMPSON, W. J., STRADA, S. J., COUTURE-MURILLO, B., MORRIS, H. P. & ROBISON, G. A. (1977) Comparison of cyclic adenosine 3′,5′-monophosphate and cyclic guanosine 3′,5′-monophosphate levels, cyclases, and phosphodiesterases in Morris hepatomas and liver. *Cancer Res.*, **37**, 3599.

JIMENEZ DE ASUA, L., CLINGAN, D. & RUDLAND, P. S. (1975) Initiation of cell proliferation in cultured mouse fibroblasts by prostaglandin $F_{2\alpha}$. *Proc. Natl Acad. Sci. U.S.A.*, **72**, 2724.

LEFFERT, H. L., KOCH, K. S. & RUBACALVA, B. (1976) Present paradoxes in the environmental control of hepatic proliferation. *Cancer Res.*, **36**, 4250.

LOWRY, O. H., ROSEBROUGH, H. J., FARR, A. L. & RANDALL, R. J. (1951) Protein measurement with the folin phenol reagent. *J. Biol. Chem.*, **193**, 265.

McMANUS, J. P., FRANKS, D. J., YOUDALE, T. & BRACELAND, B. M. (1972) Increases in rat liver cyclic AMP concentrations prior to the initiation of DNA synthesis following partial hepatectomy or hormone infusion. *Biochem. Biophys. Res. Commun.*, **49**, 1201.

PASTAN, I. H., JOHNSON, G. S. & ANDERSON, W. B. (1975) Role of cyclic nucleotides in growth control. *Annu. Rev. Biochem.*, **44**, 491.

RICHARDSON, U. I., TASHIAJAN, A. H. & LEVINE, L. (1969) Establishment of a clonal strain of hepatoma cells which secrete albumin. *J. Cell Biol.*, **40**, 236.

RYAN, W. L. & HEIDRICK, M. L. (1974) Role of cyclic nucleotides in cancer. In *Advances in Cyclic Nucleotide Research*, Vol. 4, Eds. P. Greengard & G. A. Robison. New York: Raven Press, p. 81.

SALOMON, Y., LONDOS, C. & RODBELL, M. (1974) A highly sensitive adenylate cyclase assay. *Anal. Biochem.*, **58**, 541.

SHORT, J., TSUKADA, K., RUDERT, W. A. & LIEBERMAN, I. (1975) Cyclic adenosine 3′,5′-monophosphate and the induction of deoxyribonucleic acid synthesis in liver. *J. Biol. Chem.*, **250**, 3602.

SYKES, J. A. (1976) Significance of prostaglandins in tumor growth. In *Chemotherapy*, Vol. 7, Eds. K. Hellman and T. Connors. New York: Plenum Press, p. 83.

THOMAS, E. W., MURAD, F., LOONEY, W. B. & MORRIS, H. P. (1973) Adenosine 3′,5′-monophosphate and guanosine 3′,5′-monophosphate: concentrations in Morris hepatomas of different growth rates. *Biochem. Biophys. Acta*, **297**, 564.

TOMASI, V., RETHY, A. & TREVISANI, A. (1973) Soluble and membrane-bound adenylate cyclase activity in Yoshida ascites hepatoma. *Life Sci*, **12**, Part II, 145.